# Причины рецидива ожирения после снижения массы тела

**DOI:** 10.14341/probl13275

**Published:** 2023-11-02

**Authors:** Р. М. Гусейнова, А. В. Доровских, О. В. Васюкова, Е. А. Шестакова, П. Л. Окороков, Н. Г. Мокрышева

**Affiliations:** Национальный медицинский исследовательский центр эндокринологии; Национальный медицинский исследовательский центр эндокринологии; Национальный медицинский исследовательский центр эндокринологии; Национальный медицинский исследовательский центр эндокринологии; Национальный медицинский исследовательский центр эндокринологии; Национальный медицинский исследовательский центр эндокринологии

**Keywords:** ожирение, рецидив ожирения, обмен веществ, адаптивный термогенез, гормоны, голод, аппетит, диета

## Abstract

Ключевая проблема при лечении пациентов с ожирением — отсутствие долгосрочного результата, достигнутого при снижении массы тела. С одной стороны, это объясняется низкой комплаентностью пациентов и отсутствием самоконтроля в питании и физической активности, с другой — многочисленными физиологическими механизмами, не поддающимися осознанному контролю. Текущие исследования показывают, что попытки снижения массы тела стимулируют активацию адаптационных биологических механизмов, препятствующих дальнейшему ее снижению.Несмотря на существующее множество методов лечения, лишь небольшое число пациентов способны достичь значимого (хотя бы 5–7%) снижения массы тела и поддерживать результат в дальнейшем. В большинстве случаев наблюдается возврат к исходным параметрам в среднем за 3–5 лет. Изучение механизмов, способствующих обратному набору массы тела, требует детального изучения для определения новых эффективных стратегий лечения ожирения.В обзоре литературы представлены результаты современных исследований взаимосвязи центральных, периферических и поведенческих патогенетических механизмов, приводящих к рецидиву ожирения, а также предложения по будущим стратегиям их решения.

## Abstract (en)

The main problem of obesity treatment is the difficulty of long-term weight maintenance. From one point of view, it can easily be explained by patients’ low compliance and absence of self-control. From another point of view, body weight is regulated not only by persons will, but also by multiple physiological mechanisms. Moreover, studies demonstrate that the attempts to reduce body weight stimulate the activation of adaptive biological process that block weight reduction.

Despite the variety of obesity treatment methods, only few patients are able to achieve significant (at least 5–7%) weight loss and maintain the result. In most cases people return to the initial weight in about 3–5 years. Therefore it is relevant to study weight regain mechanisms in order to identify new effective obesity treatment strategies.

The objective of this review is to summarize the information about the main issues of central, peripheral and behavioral pathogenic mechanisms which lead to disease relapse after obesity treatment and ideas for future strategies to resolve them.

## Поиск и критерии отбора литературных источников

При подготовке обзора были использованы следующие полнотекстовые и библиографическо-реферативные базы данных: Национальной медицинской библиотеки США (PubMed, Medline, Google Scholar); научной электронной библиотеки eLIBRARY.RU и КиберЛенинка (cyberleninka.ru). Поиск источников первичной информации осуществлялся на глубину 27 лет (1995–2022 гг.) по следующим ключевым словам (в англоязычных базах данных — с соответствующим переводом): ожирение; рецидив ожирения; обмен веществ; адаптивный термогенез; гормоны; голод; аппетит; диета. Сайты издательств Springer, Elsevier и Oxford Academic использовались для доступа к полному тексту статей. Для повышения специфичности и чувствительности поиска использовались логические операторы (AND OR) и фильтры: типы статей — клинические исследования, систематические обзоры, книги, метаанализы.

## Введение

Ожирение — хроническое прогрессирующее заболевание с тенденцией к рецидиву. В ряде исследований показано, что уже в течение первого года после снижения веса масса тела может достигать исходных значений [1][2]. В метаанализе 29 долгосрочных исследований более 50% потерянного пациентами веса было восстановлено в течение первых двух лет и более 80% — в течение пяти лет [3].

К настоящему времени механизмы рецидива заболевания недостаточно изучены. На фоне снижения массы тела отмечается биологическая адаптация различных систем, направленная на возврат к исходному весу. Так, снижение мотивации, несоблюдение рекомендаций по питанию и физической активности с одной стороны, а также неконтролируемые волей метаболические, нейроэндокринные и вегетативные адаптивные реакции — с другой, противостоят поддержанию достигнутых результатов [4–7].

Чаще всего после начала мероприятий по похудению вес интенсивно снижается, однако затем стабилизируется. Это объясняется тем, что изменение основного обмена, происходящее по мере снижения массы тела, ограничивает возможность поддержания адекватного темпа похудения [8]. Период стабилизации массы тела (весовое «плато») важен для получения дальнейших стойких положительных результатов. Тем не менее «плато» нередко переходит в фазу повторного увеличения веса. Такая трансформация может наблюдаться вне зависимости от выбранной тактики лечения ожирения — немедикаментозной, лекарственной или хирургической.

Данный обзор литературы посвящен описанию ключевых патофизиологических механизмов, предопределяющих рецидив ожирения.

## Влияние гормонов ЖКТ

Гормоны ЖКТ — ключевые регуляторы энергетического гомеостаза, которые действуют на центральную нервную систему (ЦНС), контролирующую прием пищи. Дефицит калорий активирует мощные компенсаторные гуморальные механизмы, препятствующие похудению. Главную роль в рецидиве ожирения играют физиологические изменения секреции гормонов ЖКТ. Так, в исследовании по оценке динамики гормонов ЖКТ после снижения массы тела на фоне 10 недель сверхгипокалорийного питания циркулирующие уровни анорексигенных гормонов (пептида YY3-36 (PYY), холецистокинина и амилина) существенно снизились по сравнению с исходным значением. Напротив, уровни орексигенных гормонов, таких как грелин, глюкозозависимый инсулинотропный полипептид (ГИП) и панкреатический полипептид (ПП), увеличились. К 52-й неделе у пациентов отмечалась тенденция к увеличению массы тела, однако гормональный дисбаланс и усиленное чувство голода сохранялись [9]. В исследовании, проведенном среди пациентов с ожирением в течение 8 недель в группе, придерживающихся низкокалорийной диеты со средней потерей веса 13% и последующей 52-недельной программой поддержания веса, снижение массы тела ассоциировалось с высокими уровнями циркулирующих постпрандиальных PYY и глюкагоноподобного пептида-1 (ГПП-1) [10]. Существуют данные, что у пациентов с ожирением и у лиц с нормальным индексом массы тела (ИМТ) при снижении веса наблюдается увеличение уровня грелина и усиление чувства голода. Постпрандиальные концентрации ГПП-1, общего PYY и холецистокинина оставались низкими у людей с ожирением в течение всего периода наблюдения по сравнению с контрольной группой [11]. Аналогичные изменения были продемонстрированы в других исследованиях [12–14]. Эти данные говорят о том, что даже длительное сохранение веса после его исходного снижения не приводит к адаптации секреции гормонов ЖКТ.

Эффект влияния гормонов ЖКТ на массу тела применяется на практике: инкретиновая терапия с использованием агонистов рецепторов ГПП-1 зарекомендовала себя в лечении ожирения. Данная терапия модернизируется, двойные и тройные агонисты рецепторов инкретиновых гормонов представляют собой перспективные препараты для лечения ожирения. Новые препараты связываются не только с рецепторами ГПП-1, но и с рецепторами ГИП и/или глюкагона, их эффект направлен на одновременное воздействие на различные метаболические пути углеводного, липидного и белкового обменов [15–17].

Доказано, что терапия агонистами ГПП-1 способствует не только снижению массы тела, но и ее удержанию. В исследовании с применением лираглутида 3,0 мг у пациентов отмечалось значительное уменьшение чувства голода и увеличение чувства сытости. В контрольной группе испытуемых на интенсивной поведенческой терапии без использования лираглутида 3,0 мг отмечено снижение чувства сытости, что привело к меньшей динамике массы тела [18][19].

Таким образом, гормоны ЖКТ являются частью сложнейшей цепи физиологического регулирования веса, и нарушение регуляции их секреции патогенетически связано с развитием и поддержанием избыточной массы тела. Несмотря на эффективное похудение на фоне различных воздействий, немедикаментозная коррекция веса зачастую встречает активное «сопротивление» организма, тогда как применение препаратов, основанных на действии гормонов ЖКТ, позволяет поддерживать достигнутый результат.

## Регуляция аппетита, голода и насыщения

Центральная регуляция пищевых поведенческих реакций реализуется за счет взаимодействия гипоталамической и мезокортиколимбической систем [20–22]. «Центр насыщения» представлен вентромедиальным ядром гипоталамуса, «центр голода» — аркуатным и паравентрикулярными ядрами, латеральной гипоталамической областью и ядром одиночного пути. Указанные структуры отвечают за синтез и рецепцию орексигенных (нейропептид Y (НПY) и агутиподобный белок (АПБ), стимулирующие прием пищи) и анорексигенных факторов (проопиомеланокортин (ПОМК) и кокаин-амфетамин-регулируемый транскрипт (КАРТ), подавляющие аппетит) (рис. 1) [20][23][24].

**Figure:**
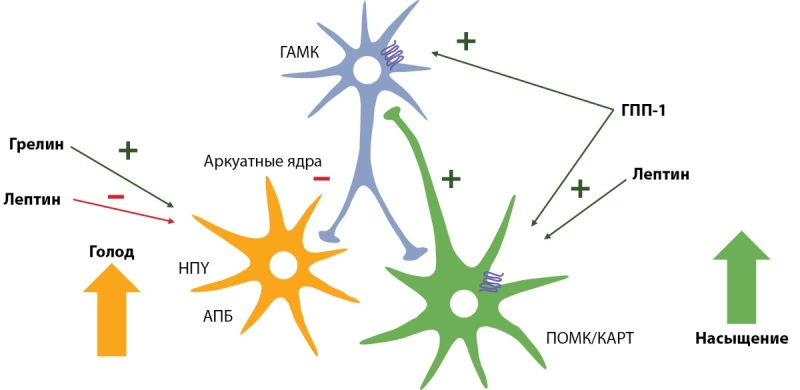
Рисунок 1. Схематичное представление центральной регуляция аппетита и голода (ориг).

Ограничение калорийности рациона приводит к снижению уровней лептина, холецистокинина и повышению грелина. При этом в дугообразных ядрах гипоталамуса повышается выработка анаболических нейромедиаторов НПY и АПБ, а также снижается синтез катаболических нейромедиаторов ПОМК и КАРТ [25–27]. Данные преобразования лежат в основе повышения аппетита на фоне снижения массы тела.

Важная роль в регуляции пищевого поведения отводится также влиянию нейротрансмиттеров, а именно дофамину и серотонину. В ряде исследований показано, что пациенты с ожирением имели дисфункцию как серотонин-, так и дофаминергической систем [28]. Серотонин участвует в регуляции энергетического гомеостаза, оказывая влияние на пищевое поведение путем стимуляции центра пищевого насыщения и торможения центра голода. При ожирении наблюдается дисфункция серотонинергической системы, что приводит к изменению пищевого поведения, дополнительным приемам пищи с высоким содержанием углеводов, являющейся наиболее сильным стимулятором синтеза серотонина в ЦНС, и как следствие — еще большему набору массы тела [22]. Дофаминергические пути участвуют в формировании двигательной активности, мотивации и эмоциональных реакций, получении удовольствия. Дофамин также является важной частью «системы вознаграждения» ЦНС. Повышенное потребление продуктов с высоким содержанием жиров и углеводов приводит к увеличению уровня дофамина, что стимулирует прием большего количества пищи [22].

Способом влияния на активность нейромедиаторов при ожирении с целью удержания массы тела является назначение препаратов центрального действия, таких как сибутрамин, комбинации фентермин/топирамат*, налтрексон/бупропион*. На сегодня оценка концентрации и эффектов как гормонов ЖКТ, так и нейромедиаторов, при изменении массы тела носит научный характер, в связи с чем индивидуализация подходов к удержанию веса, по данным гормональных исследований, затруднительна. Однако существуют практические способы контроля процесса снижения массы тела, основанные на определении основного обмена и композиционного состава тела.

## Адаптивный термогенез

Основной обмен составляет до 60–70% суточных энерготрат и характеризует расход энергии, необходимый для поддержания работы жизненно важных функций организма (дыхание, кровообращение и др.) в состоянии покоя [29]. Интенсивность основного обмена снижается с возрастом и имеет гендерные различия. Для мужчин характерны более высокие абсолютные значения базального метаболизма по сравнению с женщинами, что обусловлено особенностями композиционного состава тела. В настоящее время доказано, что главным фактором, определяющим до 70% вариабельности основного обмена, в том числе у лиц с ожирением, является тощая масса [30]. К другим факторам, влияющим на скорость базального метаболизма, относятся гормональный статус (тиреоидные и половые гормоны, лептин), активность симпатической нервной системы и генетическая предрасположенность [5][4][31].

Уменьшение калорийности суточного рациона, широко используемое в рамках лечебных стратегий по снижению веса, запускает механизмы «адаптивного термогенеза» (АТ), направленные на максимально эффективное использование организмом энергетических субстратов и противодействующих дальнейшей редукции массы тела [6][32].

На метаболическом уровне АТ реализуется за счет снижения интенсивности основного обмена и уменьшения энерготрат на физическую активность. Последний компонент вносит основной вклад в снижение суточных энергортрат у лиц с «отрицательным энергетическим балансом», уменьшая расход энергии на физическую активность на 10–35% [33]. Происходит это за счет изменения активности митохондриального окисления, соотношения гликолитических и окислительных ферментов в скелетной мускулатуре, снижения интенсивности липогенеза. Подобные механизмы метаболической адаптации уменьшают энергетическую ценность мышечных сокращений, что может замедлять темпы снижения веса даже при условии сохранения прежнего объема и характера двигательной активности [34].

К настоящему времени важность АТ в качестве ключевого механизма, способствующего восстановлению веса, остается предметом дискуссий в связи с неоднозначностью получаемых в ходе исследований данных. Так, в ряде краткосрочных исследований описано незначительное изменение основного обмена при диетиндуцированном похудении [35–38]. В то же время ряд исследований показывают достоверную связь компенсаторного уменьшения скорости основного обмена в ответ на снижение массы тела, как в краткосрочном периоде на фоне диетотерапии и расширения физической активности, так и при динамическом наблюдении в течение года [6][39]. В одном из исследований сообщается о длительном (в течение 6 лет) сохранении механизмов АТ у лиц с ожирением, что необходимо учитывать при планировании долгосрочных лечебных стратегий [40].

Практический подход к удержанию массы тела основывается на контроле композиционного состава тела в совокупности с определением параметров основного обмена. Обычно, несмотря на то, что главной целью программ по снижению веса является уменьшение количества жировой ткани, у большинства пациентов отмечается сопутствующее снижение тощей массы. Умеренное снижение количества тощей массы на фоне снижения веса является физиологичным и связано с уменьшением нагрузки на скелетную мускулатуру. Однако при сохраняющейся гиподинамии или дефиците белка в суточном рационе может отмечаться более выраженная потеря тощей массы, которая определяется с помощью методов оценки композиционного состава тела. Снижение доли тощей массы будет приводить к снижению интенсивности основного обмена.

Важно объяснять пациентам, что ограничение калорийности питания без увеличения физической нагрузки приводит к снижению мышечной массы и уровню основного обмена, что препятствует дальнейшему похудению.

## Мотивационные и поведенческие аспекты

В дополнение к вышеописанным физиологическим процессам существуют также поведенческие и психологические факторы, влияющие на поддержание массы тела.

Эволюционно сформированы и эффективно функционируют мотивационные процессы, предопределяющие поведение, направленное на потребление пищи. Соответственно, при отсутствии должного когнитивного контроля это может привести к потреблению еды сверх метаболических потребностей [41]. Гедонистическое восприятие еды подавляет сигналы сытости, стимулируя потребление вкусной пищи даже тогда, когда человек не чувствует голода, что подрывает способность к самоконтролю, особенно в современных условиях постоянной доступности пищи [42]. В исследованиях поведения лиц с ожирением повышенная тяга к еде была прямо пропорциональна ИМТ, особенно на фоне предшествующего соблюдения ограничительной диеты [43–45]. Такая биологическая особенность у лиц с ожирением приводит к особому пищевому поведению, выражающемуся в повышенном влечении к употреблению пищи.

Самоконтроль рациона питания, физической активности и массы тела — одни из основных поведенческих стратегий в борьбе с ожирением. Однако со временем приверженность пациентов к самоконтролю снижается, что и ассоциировано с рецидивом [46–48]. В исследованиях те участники, которые придерживались самоконтроля во время длительного лечения, продолжали терять вес, в то время как у других отмечен обратный набор массы тела после успешного снижения [46]. Низкая приверженность к самоконтролю наиболее часто наблюдается среди пациентов с морбидным ожирением, в связи с чем с целью эффективного лечения необходим междисциплинарный подход с привлечением психологов и психиатров. Это может помочь снизить риск психологического стресса, а также улучшить качество жизни пациентов [47].

Существует множество различных психологических подходов к терапии пациентов с ожирением, к которым относится психоанализ, гештальт, понимающая терапия, терапия принятием и ответственностью. Наиболее обоснованной и обладающей доказанной эффективностью в работе с аффективными расстройствами и нарушениями пищевого поведения является когнитивно-поведенческая терапия. Когнитивая модель базируется на представлении о том, что в основе дезадаптивного поведения человека и его психологических расстройств (нарушения пищевого поведения, режима сна и питания, отсутствие физической активности) лежит дисфункциональное мышление [49].

Это очень характерно для пациентов с ожирением, для которых типична такая деструктивная реакция, как употребление пищи в момент психологической потребности. Данный подход позволяет в относительно краткосрочном периоде обеспечить решение проблемы и проводить постепенное обучение пациента самопомощи.

## ЗАКЛЮЧЕНИЕ

Продолжающийся глобальный рост распространенности ожирения и ограниченное количество эффективных и безопасных методов лечения являются одной из проблем современного здравоохранения. В ответ на снижение массы тела происходит запуск неподвластных преднамеренному контролю компенсаторных механизмов, которые стимулируют избыточное потребление пищи, снижение расхода энергии, и, как следствие, увеличение массы тела (рис. 2).

**Figure:**
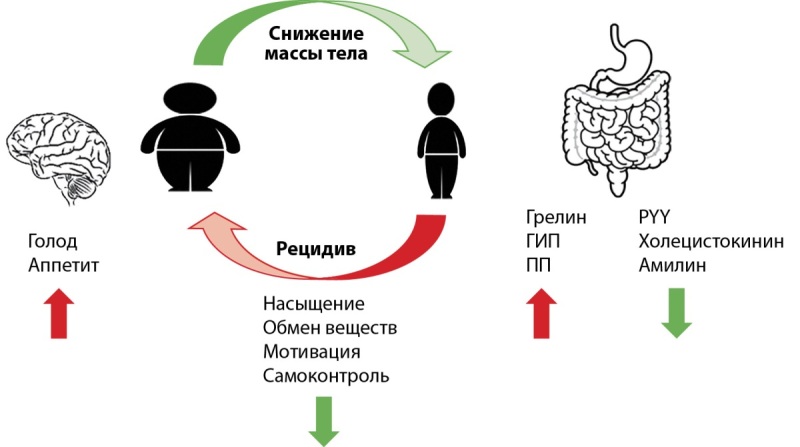
Рисунок 2. Изменения при снижении массы тела у лиц с ожирением (ориг). Комментарий. При снижении массы тела секреция орексигенных гормонов в ЖКТ, как правило, увеличивается, в то время как выработка анорексигенных замедляется. О потере жировой массы сигнализирует снижение уровня лептина. Воздействие данных сигналов на уровне нейронов гипоталамуса приводит к усилению чувства голода, аппетита и снижению энергозатрат, создавая порочный круг активизации центральных механизмов.

Работа по снижению и удержанию веса у пациентов с ожирением может быть максимально эффективной в междисциплинарной программе модификации образа жизни, которая будет направленна на изменение пищевого поведения, физической активности и поддержание приверженности наиболее эффективным медикаментозным и психологическим методам коррекции веса.

## ДОПОЛНИТЕЛЬНАЯ ИНФОРМАЦИЯ

Источники финансирования: НИОКТР 123021300168-7.

Конфликт интересов. Авторы декларируют отсутствие явных и потенциальных конфликтов интересов, связанных с содержанием настоящей статьи. Н.Г. Мокрышева является членом редакционной коллегии журнала «Ожирение и метаболизм».

Участие авторов. Гусейнова Р.М. — поиск литературы, анализ литературных данных, написание статьи; Доровских А.В. — поиск литературы, анализ литературных данных, написание статьи; Шестакова Е.А. — поиск литературы, редактирование рукописи, внесение правок; Васюкова О.В. — редактирование рукописи, внесение правок; Окороков П.Л. — поиск литературы, анализ литературных данных, внесение правок; Мокрышева Н.Г. — финальная редакция статьи.

Все авторы одобрили финальную версию статьи перед публикацией, выразили согласие нести ответственность за все аспекты работы, подразумевающую надлежащее изучение и решение вопросов, связанных с точностью или добросовестностью любой части работы.

* не зарегистрированы в РФ
